# Association of environmental and sociodemographic factors with life satisfaction in 27 European countries

**DOI:** 10.1186/s12889-019-6886-y

**Published:** 2019-05-10

**Authors:** Nikita B. Rajani, Vassilis Skianis, Filippos T. Filippidis

**Affiliations:** Department of Primary Care and Public Health, School of Public Health, Imperial College London, London, UK

**Keywords:** European Union, Life satisfaction, Weather, Environment

## Abstract

**Background:**

Life satisfaction is a cognitive and evaluative judgement of one’s outlook on life and an integral component of subjective wellbeing. There is a strong association between life satisfaction and mental and physical health, but it is currently unclear how environmental factors may influence life satisfaction. Our aim was to investigate the association between environmental factors and life satisfaction and to gain a better understanding of general life satisfaction statistics in the EU.

**Methods:**

We used a three-level mixed effects logistic regression model to examine the effects of sociodemographic, macroeconomic and environmental factors on life satisfaction using a large sample size from Eurobarometer surveys (*n* = 268,696) representative of 27 EU countries. Data were collected through face-to-face interviews between May 2014 and June 2015.

**Results:**

We found wide variation between countries, as well as between regions within the same country with regards to levels of life satisfaction. Having adjusted for individual sociodemographic factors, our analysis did not indicate statistically significant associations of mean temperature and precipitation with life satisfaction. However, there was a statistically significant association between environmental degradation and lower life satisfaction (OR = 0.98; 95% CI: 0.97–1.00). Consistent with existing literature, our results show statistically significant effects of sociodemographic factors such as sex, financial situation and employment on life satisfaction.

**Conclusions:**

Future research should extend analyses to a wider range of sociodemographic, macroeconomic and geographical variables to gain insight on all possible factors affecting life satisfaction to inform policy makers and ensure higher quality of life, and in turn better mental and physical health.

## Background

Life satisfaction is defined as an overall assessment of one’s attitude and feeling about their life at a certain point in time. [1] It provides a cognitive and evaluative judgement of one’s holistic outlook on life, forming an integral component of an individual’s subject wellbeing. Several studies have demonstrated that low life satisfaction is a predictor of depression, anxiety and neuroticism and has a reciprocal association with mental health problems. [2, 3] Life satisfaction has also been associated with adverse effects on physical health, mortality and morbidity. [4–7] Life satisfaction has been operationalized with single-and multiple-item measures, with single-item measures which ask individuals to evaluate their life satisfaction as a whole using a numerical scale performing similarly to multiple-item scales. [8, 9]

Life satisfaction is an important indicator of one’s wellbeing and is associated with mental and physical health. Understanding its correlates and determinants can provide further insights into these associations and into ways that life satisfaction measures can be used in public health research and practice. Although there is strong evidence which suggests that being employed, educated, married and financially stable are all positively associated with life satisfaction, [10–14] such investigations are of relatively limited scope as they focus on individual factors. Research on ‘external’ factors such as the environment, the weather and climate which may affect entire populations has provided less consistent findings. Although the consensus is that environmental factors can influence life satisfaction, findings for individual factors such as green space, air pollution, urban environments and overall “natural capital” have been mixed. [15–19] Regarding weather, some studies have found associations between short-term weather characteristics and life satisfaction, including reporting higher life satisfaction on sunny days compared to rainy days, [20] a positive association between sunny weather and life satisfaction, and a negative association between rain and life satisfaction. [21] However, one of the biggest studies with a sample of more than one million individuals found little evidence of an association between weather conditions and life satisfaction in the United States. [9] Despite some studies having reported associations of life satisfaction with long-term climate factors, such as precipitation and sunshine hours, [22, 23] it seems that it may be the short-term weather variability rather than the climate that affects life satisfaction. [24]

Many of the previous studies on the topic have used data from one country at a time [20, 21, 24, 25]. This may have limited the variation in weather and environmental conditions assessed and could partly explain the discrepancies between studies. Others have focused on climate using country-level data on life satisfaction score averages. [26, 27] To the authors’ knowledge only a handful of studies have examined the topic using both individual and country-level data [28, 29] The advantages of examining the impact of environmental variables on individual (micro-level) life satisfaction across several countries (macro-level) are large sample sizes and variation in both life satisfaction and weather at the country-level. Disentangling these associations can contribute to the understanding of the broader determinants of life satisfaction, which in turn will allow researchers to effectively control for these factors when exploring associations with other key variables. Therefore, we conducted an analysis of Eurobarometer data from 27 European Union (EU) countries, with the aims to contribute to existing literature on the association between environmental factors and life satisfaction and to gain a better understanding of general life satisfaction statistics in the EU. Furthermore, we took advantage of the multinational sample to examine the associations of several sociodemographic and macroeconomic variables with life satisfaction.

## Methods

### Data sources

Our analysis was based on publicly available Eurobarometer Survey data collected from 28 EU member states from June 2014 to May 2015. [30] Eurobarometer surveys are conducted in several waves every year and each wave includes a number of core questions and bespoke modules in a wide range of topics. In recent years, a question on life satisfaction has been added to the core Eurobarometer questionnaire. For the purpose of this analysis, we analysed data in waves 81.4 (data collected in May/June 2014; *n* = 28,004); 81.5 (June 2014; *n* = 27,910); 82.1 (September 2014; *n* = 28,050); 82.2 (October 2014; *n* = 27,868); 82.3 (November 2014; *n* = 27,901); 82.4 (November/ December 2014; *n* = 27,801); 83.1 (February/March 2015; n = 27,980); 83.2 (March 2015; n = 28,082); 83.3 (May 2015; *n* = 27,758); and 83.4 (May/June 2015; n = 27,718). These waves were selected in order to obtain data collected during a 12-month period.

Data was collected through personal interviews in the respondents’ language from a total of 279,092 individuals aged 15 years and older; however, some region-level data were not available for Croatia, hence it was excluded from the analysis; the final sample size was 268,696. A multi-stage random sampling and post stratification weighting were used to ensure samples are representative in terms of age, gender and area of residence. Sampling design and interview protocols were consistent across waves. Eurobarometer reports the country and Nomenclature of Units for Territorial Statistics (NUTS) region of residence. NUTS-2 level detail is available for all countries with the exceptions of Germany and the United Kingdom, where data at the NUTS-1 level was recorded. NUTS is a geocode standard to reference the subdivisions of EU countries, with NUTS-1 representing major socio-economic regions or government office regions and NUTS-2 representing basic regions for the application of regional policies, counties or groups of counties. [31]

The Climatemps database was used to obtain data on weather variables, [32] while macroeconomic and region-level aggregate data were downloaded from the Eurostat database. [33] All data were publicly available and deidentified, hence no ethical approval was required.

### Measures

#### Life satisfaction

Life satisfaction was measured using a one-item measure which asked participants, “On the whole, are you very satisfied, fairly satisfied, not very satisfied or not at all satisfied with the life you lead?” We used the original responses for our main analysis. A sensitivity analysis with an alternative grouping of responses (“very satisfied” vs. “fairly satisfied”, “not very satisfied” and “not at all satisfied”) was also conducted.

#### Sociodemographic factors

The Eurobarometer survey also collected data on participants’ age (15–24, 35–34, 35–44, 45–54, 55–64, 65+ years), sex (male, female), occupation (employed, house person, student, unemployed, retired), difficulty paying bills (never, from time to time, most of the time) as a proxy of financial difficulties, marital status (single, married, divorced, widowed, other) and area of residence (urban, rural).

#### Environmental factors

Our analysis includes annual precipitation (presented per 100 litres per square meter/100 L/m^2^), sunshine (hours[h] per day), and average annual temperature (°Celsius) extracted from ClimaTemps. Weather data were matched to Eurobarometer respondents at the lowest regional level available (NUTS-1 in Germany and the United Kingdom and NUTS-2 in the remaining countries). For each NUTS region, the capital -or the biggest city if the capital was not available in Climatemps- was selected to represent the weather in the entire region. For 46 out of the 220 regions a direct match for climate data was not available in ClimaTemps, in which case the closest city to the capital (straight line distance) was used instead.

Data on land use with heavy environmental impact (% of total land use) was extracted from Eurostat at a regional level. Land use with heavy environmental impact includes mining and quarrying; energy production; industry and manufacturing; water and waste treatment; construction; and transport, communication networks, storage, protective works.

#### Macroeconomic factors

Data on gross domestic product (GDP) per capita (results presented per 1000 Euros) for the year 2015 was extracted from Eurostat at a regional level. To control for medium-term macroeconomic trends, we compared GDP per capita between 2008 and 2015; regions where GDP per capita decreased over this period were considered affected by the economic crisis.

### Statistical analysis

A three-level (country/region/individual) mixed effects ordered logistic regression model, allowing for clustering of observations within region and country, was used to assess the associations of life satisfaction with sociodemographic (age, area of residence, sex, financial difficulties, marital status, occupation), environmental (temperature, sunshine, precipitation) and macroeconomic factors (GDP per capita, having been affected by the crisis). The model was further adjusted for the season when data collection was conducted (summer, autumn, winter or spring). The final specification of the model was decided following considerations of collinearity and comparing alternative models using the Bayesian Information Criterion and Akaike Information Criterion. Ordered regression models account for the ordered nature of the outcome variable. The results are presented as adjusted odds ratio (OR) with 95% Confidence Intervals (95% CI) and are interpreted as the OR of reporting a higher level of life satisfaction for a one-unit change in the independent variable (or compared to a reference category in categorical independent variables). A sensitivity analysis with a multi-level logistic regression model with similar specifications was conducted to compare those who reported being ‘very satisfied’ with all other categories combined. Observations with missing responses in any of the above variables (*n* = 6122, 2.3% of total observations) were excluded from the analysis. Individuals with complete and missing data were compared using chi-square tests. Descriptive results as presented as weighted % with 95% CI. Survey weights provided in the official Eurobarometer datasets were used as appropriate to control for the complex study design. All analyses were conducted using Stata 15.0 (StataCorp LP, College Station, TX).

## Results

**Fig. 1:**
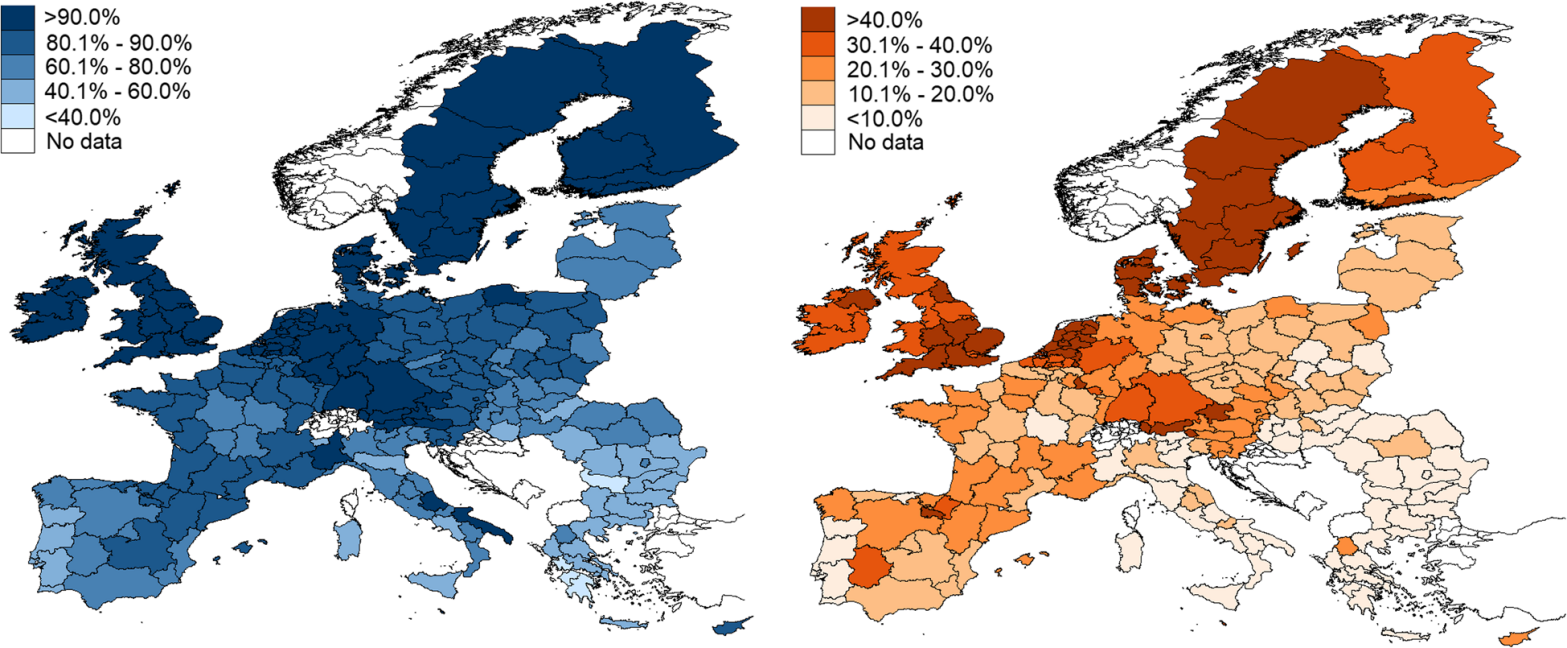
Proportion of respondents aged ≥15 years very and fairly satisfied (left) and very satisfied (right) with their lives by NUTS-1 and NUTS-2 region in the European Union, 2014–2015 (map created by the authors with Stata 15.0)

Table 1 provides an overview of the population aged over 15 years old who are satisfied (very and fairly satisfied) and very satisfied with life across the 27 EU countries, while Fig. 1 shows the proportion of satisfied and very satisfied individuals at a regional level. Denmark (97.9%) and Sweden (96.3%) have the highest proportion of satisfied population. Overall, countries with the highest proportions of satisfied respondents are in Western Europe. The only exception is France with a somewhat lower proportion (83.7%). Interestingly, the countries of the South (Greece, Italy, Portugal, and Spain) appear to have lower levels of satisfaction: 46, 65.1, 56 and 77.2% of the population is satisfied with life respectively. Greece has the lowest levels of self-reported life satisfaction among all EU countries. In addition, Greece and Bulgaria are the only countries where satisfied citizens are the minority − 46 and 46.7% for Greece and Bulgaria respectively. Relatively low levels of life satisfaction, ranging between 60.6 and 78.4%, were also reported in some Eastern European countries, including Estonia, Latvia, Lithuania, Hungary and Romania.

**Table 1:** Proportion of the population aged 15 years and above who are very and fairly satisfied with their life in 27 EU countries, 2014–2015

Country	Very and fairly satisfied		Very satisfied	
	%	95% CI	%	95% CI
France	83.7	82.9–84.5	20.4	19.5–21.3
Belgium	89.6	88.9–90.2	27.7	26.7–28.7
The Netherlands	95.1	94.6–95.5	52.4	51.4–53.5
Germany	90.0	89.4–90.5	29.1	28.2–29.9
Italy	65.1	64.1–66.1	7.0	6.5–7.6
Luxembourg	94.9	94.1–95.5	40.6	39.1–42.1
Denmark	97.9	97.6–98.2	71.7	70.7–72.7
Ireland	92.0	91.4–92.5	38.2	37.2–39.2
United Kingdom	92.3	91.8–92.5	40.5	39.5–41.5
Greece	46.0	45.0–47.0	5.6	5.2–6.1
Spain	77.2	76.3–78.0	19.8	19–20.6
Portugal	56.0	55.0–57.0	3.7	3.3–4.1
Finland	94.4	93.9–94.9	37.3	36.2–38.3
Sweden	96.3	95.8–96.7	48.2	47.0–49.4
Austria	89.9	89.2–90.5	32.8	31.8–33.8
Cyprus (Republic)	82.8	81.7–83.9	27.7	26.4–29.0
Czech Republic	83.3	82.6–84.0	17.3	16.5–18.1
Estonia	78.4	77.5–79.3	12.0	11.3–12.8
Hungary	64.0	63.1–65.0	8.9	8.3–9.5
Latvia	71.0	69.9–72.0	11.8	11.0–12.5
Lithuania	73.1	72.2–74.0	15.4	14.7–16.2
Malta	91.9	91.0–92.7	37.5	35.9–39.0
Poland	83.6	82.8–84.4	13.4	12.7–14.2
Slovakia	72.7	71.8–73.6	14.7	13.9–15.5
Slovenia	84.9	84.1–85.6	25.7	24.8–26.6
Bulgaria	46.7	45.7–47.7	4.0	3.6–4.4
Romania	60.6	59.6–61.5	8.0	7.4–8.5

Multi-level regression results are presented in Table 2. In the main analysis our model has not detected any statistically significant correlation between climate variables, i.e. mean annual temperature, sunshine hours and precipitation, and life satisfaction. However, environmental degradation was statistically significantly associated (OR = 0.98; 95% CI: 0.97–1.00 for a 1-unit increase in land use with heavy environmental impact as % of total land) with reporting lower levels of life satisfaction. This finding was not statistically significant in the sensitivity analysis. The odds of reporting satisfaction with life were highest during spring.

**Table 2:** Multi-level regression exploring the association of sociodemographic, macroeconomic and environmental variables with life satisfaction in 27 EU countries, 2014–2015

	Ordered regression model^a^			Logistic regression model^b^		
	Odds Ratio	*p*-value	95% CI	Odds Ratio	*p*-value	95% CI
Area of residence						
Rural (ref.)						
Urban	1.00	0.597	0.98–1.01	0.98	0.098	0.96–1.00
Gender						
Female (ref.)						
Male	0.91	< 0.001	0.89–0.92	0.92	< 0.001	0.90–0.94
Marital status						
Married (ref.)						
Single	0.66	< 0.001	0.64–0.68	0.66	< 0.001	0.64–0.68
Divorced	0.58	< 0.001	0.56–0.59	0.59	< 0.001	0.56–0.61
Widowed	0.60	< 0.001	0.58–0.62	0.61	< 0.001	0.58–0.63
Other	0.74	< 0.001	0.67–0.82	0.74	< 0.001	0.66–0.83
Occupation						
Employed (ref.)						
Houseperson	0.83	< 0.001	0.80–0.86	0.94	0.008	0.89–0.98
Student	1.35	< 0.001	1.29–1.41	1.40	< 0.001	1.32–1.47
Unemployed	0.44	< 0.001	0.43–0.46	0.58	< 0.001	0.55–0.61
Retired	0.77	< 0.001	0.75–0.79	0.89	< 0.001	0.86–0.92
Age						
65+ years old (ref.)						
55–64	0.81	< 0.001	0.79–0.83	0.81	< 0.001	0.78–0.84
45–54	0.80	< 0.001	0.77–0.83	0.79	< 0.001	0.75–0.82
35–44	1.01	0.528	0.98–1.05	0.95	0.033	0.91–1.00
25–34	1.24	< 0.001	1.19–1.29	1.15	< 0.001	1.09–1.20
15–24	1.79	< 0.001	1.71–1.89	1.65	< 0.001	1.55–1.76
Difficulty paying bills						
Never (ref.)						
From time to time	0.39	< 0.001	0.38–0.40	0.38	< 0.001	0.37–0.39
Most of the time	0.14	< 0.001	0.14–0.14	0.28	< 0.001	0.27–0.30
Mean annual temperature	0.98	0.203	0.94–1.01	0.98	0.396	0.93–1.03
Sunshine (h per day)	0.94	0.171	0.87–1.03	0.93	0.204	0.83–1.04
Precipitation (per 100 L/m^2^)	1.00	0.867	0.97–1.03	1.02	0.258	0.98–1.06
GDP per capita (per €1000)	1.01	0.001	1.00–1.02	1.01	0.025	1.00–1.02
Affected by the crisis						
No (ref.)						
Yes	1.01	0.943	0.85–1.19	1.16	0.195	0.92–1.47
Land use with heavy environmental impact (as % of total land)	0.98	0.036	0.97–1.00	0.99	0.240	0.97–1.01
Season						
Spring (ref.)						
Summer	0.91	< 0.001	0.90–0.93	0.94	< 0.001	0.92–0.97
Autumn	0.97	0.013	0.96–0.99	0.96	0.002	0.94–0.98
Winter	0.94	< 0.001	0.92–0.97	0.90	< 0.001	0.87–0.94

a Results interpreted as odds ratio of reporting a higher level of life satisfaction for a one-unit change in the independent variable (or compared to a reference category in categorical independent variables)

b Results interpreted as odds ratio of being very satisfied with life -compared to all other responses- for a one-unit change in the independent variable (or compared to a reference category in categorical independent variables)

Males were less likely to report a higher level of satisfaction with their life compared to women (OR = 0.91; 95% CI: 0.89–0.92). On the other hand, the odds of reporting satisfaction with life were greater for people less than 35 years old. The association with age is particularly strong with adolescents and young adults between 15 and 24 years old (OR = 1.79; 95% CI: 1.71–1.89). Compared to those who were married, being single (OR = 0.66), widowed (OR = 0.60) or divorced (OR = 0.58) was associated with lower life satisfaction. Similarly, the odds of reporting a higher level of life satisfaction were lower for people without employment, i.e. housepersons (OR = 0.83), unemployed (OR = 0.44) or retired (OR = 0.77). The only exception is students, who were more likely (OR = 1.35) to report high levels of satisfaction. Finally, although higher GDP levels – our macro proxy for economic conditions - were associated with higher satisfaction with life, and difficulties paying bills – our micro proxy for financial conditions - were associated with lower levels of satisfaction, the economic crisis variable did not show any statistically significant associations. When comparing those with complete data to those with missing data, we found that individuals who were single, students and aged 15–24 were more likely to have been excluded from the regression analysis due to missing data.

## Discussion

We found that life satisfaction was associated with a number of individual-level factors across the EU. However, there were no significant associations between climatic factors and life satisfaction, after adjusting for the individual-level factors. Environmental degradation was inversely associated with life satisfaction, with those living in regions with extensive activities of heavy environmental impact reporting lower levels of life satisfaction.

With regards to climatic and environmental factors, empirical evidence from various studies has suggested that weather conditions are usually significant determinants of subjective well-being, mainly in the short (i.e. affecting daily mood and behavior) but in some studies also in the long-term (i.e. having an in-depth impact on human life satisfaction over time). For instance, mean annual temperature and sunshine hours have been associated with higher life satisfaction, while extreme temperatures and relative humidity have been associated with lower self-reported life satisfaction. [22, 26, 27, 34] In some cases, precipitation has been associated with higher life satisfaction levels. [29] However, some important studies in the field have not found any significant associations between weather and life satisfaction or point to only short-term influence of daily weather variation. [9, 24] The results from our analysis are consistent with the latter studies as they did not yield any statistically significant association of mean annual temperature, sunshine hours and precipitation -all elements of the local climate- with life satisfaction. Some of these discrepancies in the literature may be a result of different methodological approaches in sampling and assessing life satisfaction and weather variables. However, they might simply reflect the complexity of the association. For example, higher temperatures may be desirable in places with cold climate and undesirable during a hot summer. Additionally, high precipitation may itself be associated with lower life satisfaction, but can be linked with scenic beauty, which indirectly positively influences life satisfaction. [22]

To date, several studies mainly within the field of environmental economics and psychology have highlighted the negative effect of poor environmental conditions such as air and noise pollution on various aspects of well-being. [15, 35, 36] Our analysis is consistent with these studies as we found that respondents living in regions experiencing environmental degradation, represented by our “land use with heavy environmental impact” variable, increases the probability of reporting lower levels of satisfaction with life. Environmental degradation can be heavily influenced by public policies; our findings may motivate local and national authorities to increase efforts to protect the environment and reduce the consequences of industrial and urban activities on the environment, especially in areas with high population density.

We also found seasonal variability in life satisfaction, with respondents reporting higher levels of life satisfaction in spring. Although being more satisfied in spring compared to winter is, to some extent, intuitive, the detrimental effect of summer seems to be unexpected. One possible explanation is that a particular event or circumstances could have negatively affected people’s well-being during this particular summer period in the European Union. Additionally, extreme temperatures during the summer period are associated with lower well-being levels, particularly in countries where individuals are not accustomed to high temperatures, but could also cause events such as forest fires, which have been found to be detrimental for life satisfaction. [37] In any case, the observed association between seasonal dummies and life satisfaction cannot be directly interpreted as environmental effects, since there may be various other indirect factors that influence people’s well-being (such as outdoor activities or vacations in the summer, mountain activities in the winter, favourable or unfavourable events etc.) during these periods.

Consistent with existing literature, our findings indicate that males and unemployed individuals are more likely to report lower levels of satisfaction with life, [28, 29, 38, 39] while married individuals reported much higher satisfaction levels than single individuals. Some evidence suggests that although life satisfaction increases after marriage, it tends to decrease over time, [40] but we had no relevant data to assess this in our analysis. Moreover, our analysis indicates that having financial difficulties is associated with an increased likelihood of dissatisfaction with life, whereas higher regional GDP is associated with increased levels of life satisfaction. The latter finding adds to the debate on the relationship between GDP and subjective well-being. Although there are several studies that have found a positive relationship between GDP and subjective well-being, consensus has not been reached on whether this is always the case. [39, 41, 42] On the other hand, unfavourable macroeconomic conditions, such as inflation rate are typically linked with lower levels of well-being. [29, 43] In our analysis, we included a “crisis” dummy variable to control for countries with considerable decreases in their GDP. Although most regions with low life satisfaction were those hit by the economic crisis, the economic crisis variable was not statistically significantly associated with life satisfaction after adjusting for individual socio-demographic and environmental factors.

Although our analysis contributes to the existing literature, it is subject to some limitations. Firstly, most of the environmental variables in our analysis refer to NUTS 2 regions whilst life satisfaction and several socio-demographic variables were collected at an individual level. Weather conditions can often vary significantly even within short distances or different altitudes; therefore the region-level environmental variables may not fully reflect the climate experienced by the respondents. Secondly, we had no data on extreme weather events or natural disasters, such as storms, flood and earthquakes, which did not allow us to explore additional environmental factors. Also, we had no data on ethnicity, religion, personality traits and self-reported health, which have been identified as important indicators of subjective well-being [1, 10, 23] and missing data may have introduced selection bias. However, the proportion of individuals with missing data was relatively small and our large sample size and availability of both individual and regional data allowed us to control for major sources of confounding.

Future analyses can be extended by investigating a wider range of geographical and environmental factors, both objective and subjective. The former ones could include specific local characteristics, such as air pollution levels, proximity to coast, areas of outstanding natural beauty and heavy industries. The latter set of variables could include self-reported perceptions on the surrounding environment, nature-related activities, attitudes and behaviours towards the environment. A second potential extension of this research would be to include a wider range of countries, so that the findings can provide insight on the possible factors affecting quality of life at a global level.

## Conclusion

Our findings suggest that an interplay of sociodemographic, macroeconomic and environmental factors influence life satisfaction in the EU. They also highlight the importance of modifiable factors, such as environmental degradation and economic conditions for people’s life satisfaction. Consequently, there are plenty of opportunities for authorities and societies to positively influence life satisfaction by improving economic conditions and protecting the environment.

## Abbreviations

CI: Confidence Interval
EU: European Union
GDP: Gross Domestic Product
OR: Odds Ratio
